# Poverty, not the poor

**DOI:** 10.1126/sciadv.adg1469

**Published:** 2023-08-23

**Authors:** David Brady

**Affiliations:** ^1^ University of California, Riverside, Riverside, CA, USA.; ^2^ WZB Berlin Social Science Center, Berlin, Germany.

## Abstract

This review explains how and why the United States has systemically high poverty. Descriptive evidence shows that U.S. poverty is (i) a huge share of the population, (ii) a perennial outlier among rich democracies, (iii) staggeringly high for certain groups, (iv) unexpectedly high for those who “play by the rules,” and (v) pervasive across various groups and places. This review then discusses and critiques three prevailing approaches focused on the individual poor rather than the systemically high poverty: (i) behavioral explanations “fixing the poor,” (ii) emotive compassion “dramatizing the poor,” and (iii) cultural explanations both dramatizing and fixing the poor. The essay then reviews political explanations that emphasize the essential role of social policy generosity, political choices to penalize risks, power resources of collective political actors, and institutions. This review demonstrates a long emerging but ascending and warranted shift away from individualistic explanations of the poor toward political explanations of poverty.

## Teaser

Recent research changes our understanding of the causes of and policies for reducing systemically high poverty in the U.S.

## INTRODUCTION

The historian Michael Katz (*1*) writes, “The idea that poverty is a problem of persons—that it results from moral, cultural, or biological inadequacies—has dominated discussions of poverty for well over two hundred years and given us the enduring idea of the undeserving poor.” Scholarship and public debate about American poverty have traditionally focused on contrasting the individual poor against the individual nonpoor. For a long time, the field has highlighted the individual demographic and labor market risks that are more common among the poor. In the process, the field has emphasized the problematic choices, behaviors, cultures, and traits of the poor. It has routinely asked why the poor fail to get married, why the poor do not complete their education, and why the poor do not work. This has implied that the poor are poor because of the “problem of persons”—owing to pathological choices, behavior, cultures, and traits—and this has led to a focus on poor individuals.

This review of the field shows that these tendencies have distracted researchers from the larger and more salient issue of America’s systemically high poverty. A focus on the individual poor has been incapable of providing an accurate understanding of that systemically high poverty. Explaining the systemically high U.S. poverty requires a paradigmatic shift to focus on poverty, not the poor. This paradigmatic shift builds on a long-standing critical undercurrent in and the contested nature of poverty debates (*2*–*6*). Such a paradigmatic shift is also likely to lead to more effective anti-poverty policies.

First, this review defines the measurement of poverty. Second, it demonstrates how the United States has systemically high poverty. Third, it critically reviews three prevailing approaches focused on the problem of persons and the individual poor. Fourth, it reviews political explanations of America’s systemically high poverty. Political explanations provide a far more promising direction for American poverty research. This review culminates in showing that a long-needed paradigmatic shift is gaining momentum toward studying poverty, not the poor.

## POVERTY MEASUREMENT

Poverty is best defined as a shortage of resources compared with needs (*7*, *8*). This review, like a growing consensus of poverty researchers, explicitly avoids the deeply flawed official poverty measure (OPM) because of its well-documented validity and reliability problems (*1*, *4*, *8*–*11*). National Academy of Sciences panels in 1995 (*12*) and 2019 (*13*) both heavily critiqued the OPM. The OPM thresholds are widely understood to be too low, and the family size adjustments are incoherent. The OPM’s income definition ignores taxes and tax credits and inconsistently includes some transfers but omits others. For example, Temporary Assistance for Needy Families (TANF) and Old Age Survivor’s Insurance count as income, but the Supplemental Nutrition Assistance Program (SNAP), housing subsidies, childcare vouchers, and tax credits like the Earned Income Tax Credit (EITC) and Child Tax Credit (CTC) do not. Most of the U.S. government’s transfers to address the coronavirus disease (COVID) pandemic would be ignored by the OPM (*14*). Since the 1990s, the EITC and SNAP have grown substantially. In recent years, the CTC was substantially expanded as well. Government spending on each of SNAP, the EITC, and the CTC are now markedly larger than on TANF. Therefore, overtime comparisons based on the OPM are particularly unreliable.

Despite popular impressions, the OPM was problematic from the very beginning. The OPM is often attributed to Orshansky. However, O’Connor [(*4*), p. 184] explains, “No one was more surprised, though, than Orshansky herself, who had never meant her measures as official government standards. Concerned primarily with suggesting a way to vary the measure for family size, Orshansky took pains to recognize that her work was at best an ‘interim standard,’ ‘arbitrary, but not unreasonable,’ and minimalistic at best.” Katz [(*1*), p. 116] quotes Orshansky as writing, “‘The best that can be said of the measure,’ she wrote, ‘is that at a time when it seemed useful, it was there.’” The standard of needs underlying the OPM never had a scientific basis (*1*, *4*). Using data from the mid-1950s, Orshansky developed a rule of thumb that food amounted to roughly one-third of expenses for typical households. It was never clear that this applied to low-income households, however. Furthermore, the Johnson administration ended up using the “economy food plan,” which was about 25% below the “low-cost food budget” used by Orshansky (*1*). The economy food plan was actually only meant for emergencies and on a temporary basis. In addition, in the late 1960s, the government began updating the OPM thresholds using the consumer price index and thus severed the link to the food budget. Katz [(*1*), p. 116] quotes Orshansky as writing, “This meant, of course, that the food-income relationship which was the basis for the original poverty measure no longer was the current rationale.” Moreover, obviously unlike the mid-1950s, food is certainly far less than one-third of household expenses today. As a result, the OPM effectively ignores the increased costs of crucial needs like childcare and health care, which were less essential or much cheaper when the OPM was created.

Following the overwhelming majority of cross-national poverty research (*7*, *9*–*11*), I use a relative measure. A relative measure defines poverty as a shortage of resources relative to needs defined by the prevailing standards of a time and place. Both cross-national and U.S.-specific research show that relative measures better predict well-being, health, and life chances; are more valid for leading conceptualizations of poverty; are more reliable for overtime and cross-place comparisons; and are justified because of the absence of defensible absolute alternatives with fewer problems (*7*, *8*, *10*, *12*, *15*). The evidence in this essay mostly uses the Luxembourg Income Study (LIS) Database. The Replication File contains the entire replication code.

Following the overwhelming majority of cross-national poverty research, I also set the poverty threshold at 50% of the median equivalized “post-fisc” household income (*8*, *10*, *11*). People are poor if their income is below this threshold. Post-fisc income incorporates taxes, tax credits, and cash and near cash transfers (*7*). Income is “equivalized” by dividing by the square root of the number of household members. The poverty thresholds are established with population weights in a given year.

## SYSTEMICALLY HIGH POVERTY IN THE UNITED STATES

To say that poverty is systemically high is based on at least five patterns. U.S. poverty is (i) a huge share of the population, (ii) a perennial outlier among rich democracies; (iii) staggeringly high for certain groups, (iv) unexpectedly high even among those who “play by the rules,” and (v) pervasive across various groups and places.

### A huge share of the population

In 2019, 17.5% of the United States, about 57.4 million, was poor (*16*). Compared to more visible social problems, there are far more people in poverty. For instance, Pew Research Center (*17*) routinely surveys Americans on the biggest problems facing the nation. Among the most highly mentioned are the affordability of health care, violent crime, illegal immigration, gun violence, racism, and unemployment. There has also been considerable attention on evictions, mass incarceration, and COVID in recent years. Pew has never listed poverty as one of the biggest problems.

**Fig. 1.**
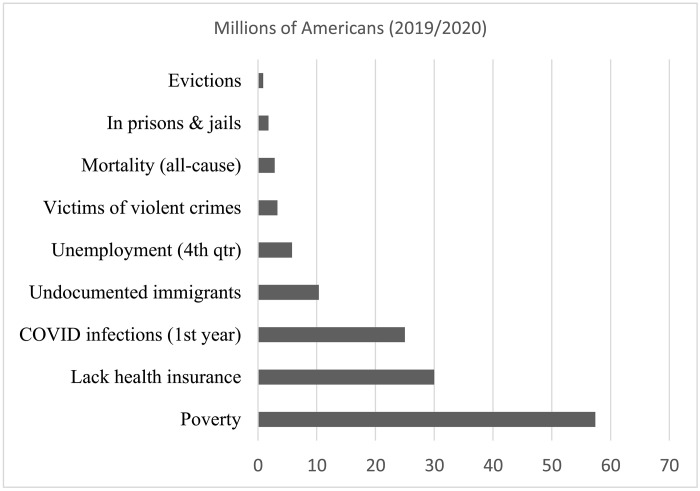
The number of millions of people in the United States experiencing major social problems (2019 or 2020). There are markedly more people in poverty in the United States than are experiencing many far more visible problems.

Figure 1 makes plain that poverty affects a huge population. Almost 64× as many people experienced poverty as experienced an eviction (*16*–*25*). More than 32× as many people were in poverty as incarcerated at one point in time. There were more than 20× as many in poverty as the number of deaths from all causes. All-cause mortality is obviously markedly larger than highly visible causes like from firearms (i.e., there is roughly one firearm-related death for every 1446 people in poverty). The population in poverty is 17× greater than the number of victims of violent crime, almost 10× the number of unemployed, and about 5.5× the number of undocumented immigrants. The number of COVID infections in the first year and the number lacking health insurance were only about half as many as in poverty.

On balance, some of these are events (e.g., evictions and mortality), while poverty and others (e.g., the incarcerated and uninsured) might be statuses, and some are both (e.g., crime is an event, but being a victim of crime is a status). People cycle in and out of poverty. Analyses with the Panel Study of Income Dynamics (PSID) and Cross-National Equivalent File reveal that about 45 to 46% of people in poverty are in their very first year of being poor (and another 19 to 25% have only been poor for 2 to 3 years) (*26*, *27*). Thus, nearly half of those with the status of poverty experience the event of falling into poverty in a year. An even larger population experiences poverty at least one point in their lives (*28*). In recent years, about 61% of Americans have experienced at least 1 year in poverty during their lives (*27*). Thus, regardless of whether it is a status or event, poverty affects a huge population.

### A perennial outlier among rich democracies

**Fig. 2.**
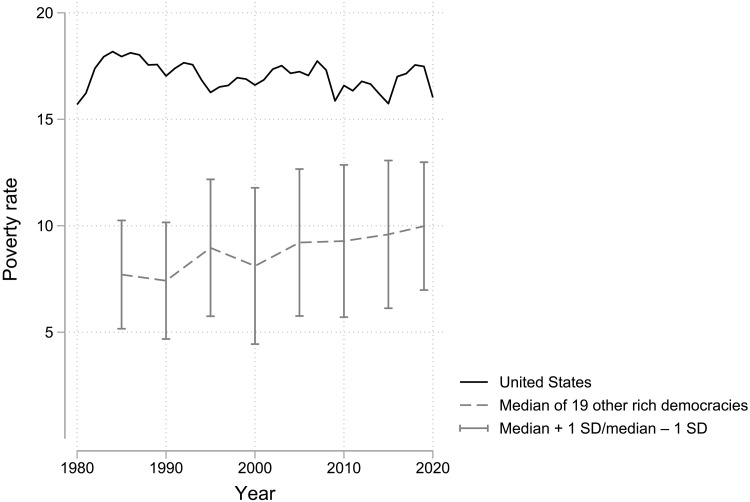
Trends in the poverty rate in the United States and for the median of 19 other rich democracies (1980–2020). For many decades, the United States has maintained consistently higher poverty compared to what is typical in other rich democracies. The solid line represents the annual poverty rate in the United States (1980–2020). The dashed line is the median poverty rate across 19 other rich democracies with data consistently available every 5 years (1985–2020). The vertical lines around the dashed line mark ± one cross-national SD in each year (*16*).

Ample research has established that the United States has high poverty compared to other rich democracies (*7*, *29*, *30*). By now, over four decades of LIS data confirm the United States has high poverty compared to Europe. What has become clear only more recently, however, is just how consistently the high U.S. poverty is an outlier among rich democracies. Figure 2 shows the trend in U.S. annual poverty rates from 1980 to 2020. The U.S. rate is consistently near its overtime average of 17% in every year. By far, the clearest “trend” in American poverty is stability at a high level. The U.S.’s stability corrects popular claims of increasing (or declining) poverty. In addition, some incorrectly claim that stability is a by-product of relative measures. This is easily contradicted by substantial overtime changes in, for example, Israel, Luxembourg, Sweden, and the United Kingdom.

Figure 2 compares the United States to the median poverty rate of 18 other peer rich democracies with LIS data every 5 years (1985–2020). The cross-national median represents “normal” or typical poverty for rich democracies. When the United States had a poverty rate near 18% in the mid-1980s, these other rich democracies had a far lower median of 7.7%. Over the entire period, the average median poverty rate in other rich democracies was only 8.8% versus 17% in the United States. Thus, the United States has maintained a poverty rate almost twice as high as peer rich democracies over the past four decades. Poverty in other rich democracies has drifted upward over time, and Israel has had higher poverty than the United States since 2005. Nevertheless, poverty in the typical rich democracy has always remained far below the United States.

### Staggeringly high for some groups

The United States carries staggeringly high poverty for some groups. As explained below, there are four major risks to poverty. The four major risk groups in the United States have staggeringly high poverty compared to those risk groups in other rich democracies (*31*). Specifically, the poverty rate for people in working-aged households where (i) no one is employed (i.e., joblessness) is 73.6%, (ii) the highest earner lacks a high school degree is 41.4%, (iii) the highest earner is under 25 years old is 31.3%, and (iv) the household is led by a single mother is 39.4%.

Beyond risk groups, the best example of staggeringly high poverty is for certain ethno-racial minorities (*32*, *33*). One conservative estimate is that Black, Latino, and Native Americans have poverty rates about twice as high as white Americans (*9*). That staggeringly high poverty skews the U.S. overall poverty rate. White and Asian Americans do not have exceptionally high poverty rates compared to most rich democracies. In turn, much of America’s systemically high poverty is simply due to staggeringly high poverty for ethno-racial minorities. To put the scale of Black poverty in perspective, one conservative estimate is that roughly 25× as many African Americans are in poverty annually as are incarcerated at one point in time (*10*).

**Fig. 3.**
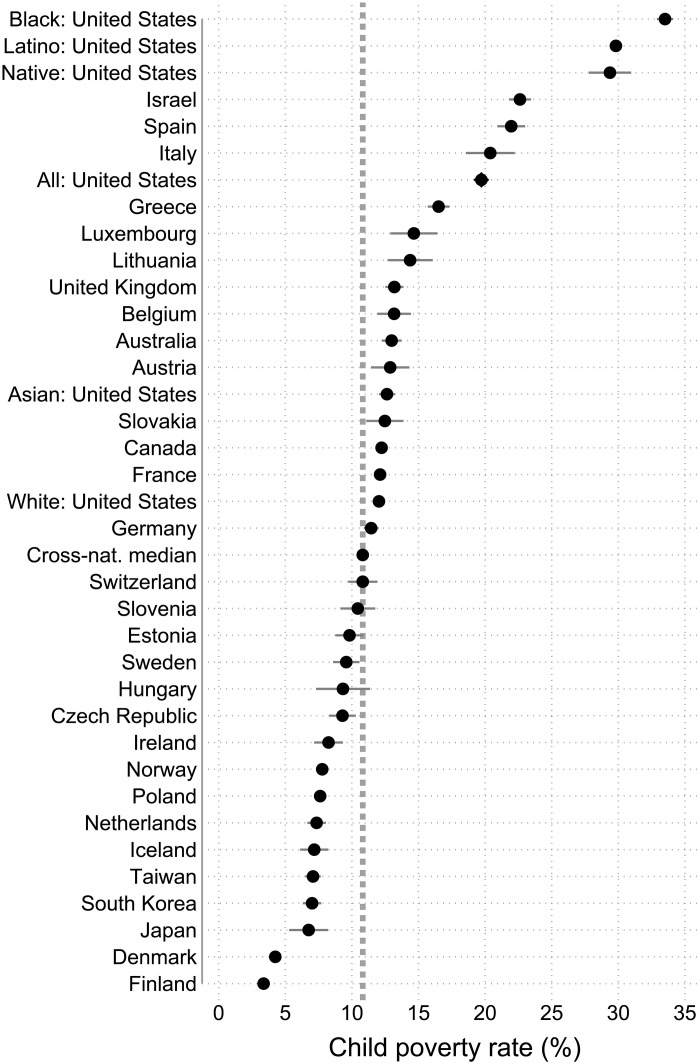
Child poverty rates for U.S. ethno-racial groups (2016–2020) and for 30 rich democracies. Poverty rates for Black, Latino, and Native American children are staggeringly high compared to child poverty rates in other rich democracies as well as Asian American and white children in the United States. By contrast, white and Asian American children exhibit poverty rates similar to children in most other rich democracies. The dots represent each group or country, and the 95% confidence intervals on those estimates are shown as spikes. The vertical dashed line is the median child poverty rate across 30 rich democracies. For the United States, estimates are based on 2016–2020 data, and, for other rich democracies, estimates are based on most recent available data (*16*).

Figure 3 illustrates this with child poverty. Black children have a poverty rate of 33.5%, Latino children are at 29.8%, and Native American children are at 29.4%. No rich democracy has anywhere near as high of child poverty rates as these three ethno-racial groups. The next highest child poverty rates are for Israel and Spain at about 22%, and no rich democracy has exceeded 26.1% in over four decades of LIS data. By contrast, white and Asian American children have poverty rates close to the cross-national median of 10.8% (vertical line). White child poverty is lower than child poverty in Canada and France. Thus, the United States has systemically high poverty partly because of staggeringly high poverty for some ethno-racial minorities alongside cross-nationally typical child poverty rates for white and Asian Americans.

**Fig. 4.**
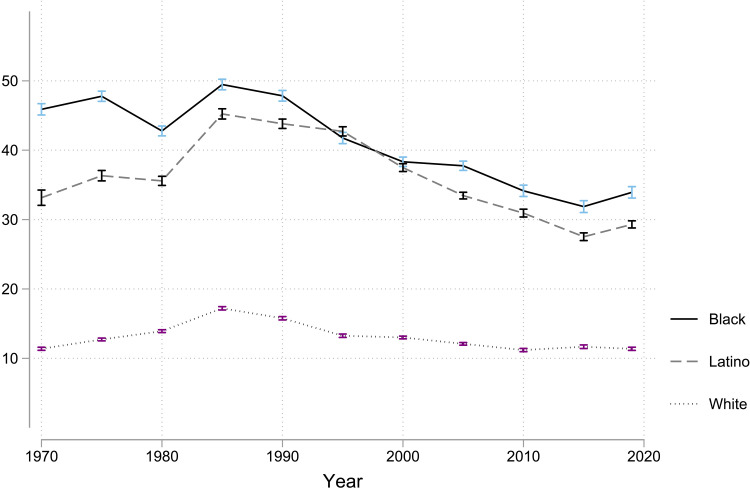
Trends in Black, Latino, and white child poverty in the United States (1970–2019). The poverty rate for Black and Latino children has consistently been markedly higher than for white children in the United States. Child poverty rates peaked in the mid-1980s, when Black and Latino child poverty rates were even more staggeringly high at near 50%. The top solid line is for Black children, the dashed line is for Latino children, and the dotted line is for white children. The 95% confidence intervals are shown as error bars (*16*).

This staggering racial inequality in poverty was even worse historically. As Fig. 4 shows, Black child poverty was near 50%, and Latino child poverty was above 45% in the 1980s. Worse, the staggeringly high Black child poverty in the 1980s was an increase compared to the 1970s (*34*, *35*). Furthermore, Black and Latino child poverty have only descended to near 30% recently. Most Black and Latino adults today grew up experiencing truly extraordinarily high poverty during childhood compared to any rich democracy in over four decades of the LIS. These child poverty rates were so overpowering that ethno-racial minority children have experienced lifelong disadvantages that both shape subsequent risks and drive high young adult poverty even net of risks (*36*).

### Unexpectedly high for those “playing by the rules”

It is well known that the four major risk groups have particularly high poverty rates in the United States (*31*). More unexpected, however, is that U.S. poverty is also quite high among people not in the risk groups. Such people have met standard expectations of socioeconomic achievement. Thus, U.S. poverty is high even among those who play by the rules in terms of work, education, marriage, and parenthood.

**Fig. 5.**
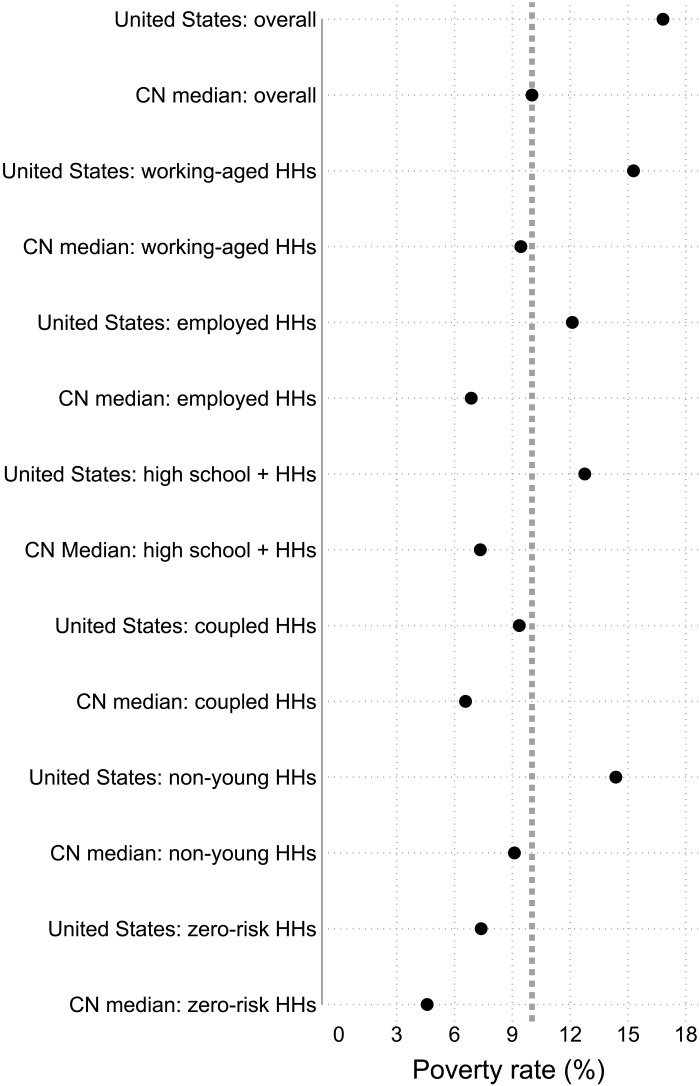
Poverty rates for the overall population and various non-risk groups in the United States (2016–2020) and at cross-national median for 31 rich democracies. Poverty rates in the United States are much higher than in other rich democracies. This is the case for the overall population, among working-aged households (HHs) and for groups that have met standard expectations of socioeconomic achievement (e.g., employment, education, marriage, age, and risks). Cross-national (CN) median is the cross-national median across 31 rich democracies. The vertical dotted line is the cross-national median poverty rate for the overall population. The dots represent poverty rates for various groups. Confidence intervals for U.S. estimates are shown as spikes but are too small to be visible (*16*).

Figure 5 shows poverty rates for non-risk groups among working-aged households (*31*). The overall poverty rate for the United States (i.e., 16.8%) and median of 31 rich democracies (i.e., 10%) are used as benchmarks. Working-aged households always have lower poverty than the overall population as poverty is higher at the ends of the life cycle. However, at 15.3%, U.S. working-aged households have a higher poverty rate than the cross-national median overall poverty rate. Employed households usually have far lower poverty because joblessness is the strongest predictor of poverty. However, employed households in the United States have a poverty rate of 12.1%, above the cross-national median. While households led by someone lacking a high school degree are obviously disadvantaged, U.S. households led by a high school graduate still have a high poverty rate of 12.8%. At 9.4%, U.S. coupled (i.e., married or cohabiting and working-aged) households have poverty rates close to the cross-national median. Non-young U.S. households (i.e., led by someone aged 25+) have a high poverty rate of 14.4%. Last, even if people meet all the mainstream expectations and have zero risks, fully 7.4% of them are in poverty.

Most Americans are not in the major risk groups. Most play by the rules. Dramatically more people are in employed (94.8%) than jobless working-aged households (5.2%). Clear majorities are in working-aged households led by someone with at least a high school degree (91.2%) and are in coupled/married households (63.0%). As a result, a substantial share of the population in poverty is not in the risk groups. The best example is that more than three times as many people are in working-poor households (9.7% of population) as jobless poor households (3.2% of population) (*37*). Hence, most of the population in poverty is similar to the U.S. population in terms of employment, age, family structure, and education. As I explain below, this severely constrains how much poverty can be reduced by reducing the size of the risk groups.

### Pervasive across various groups and places

**Fig. 6.**
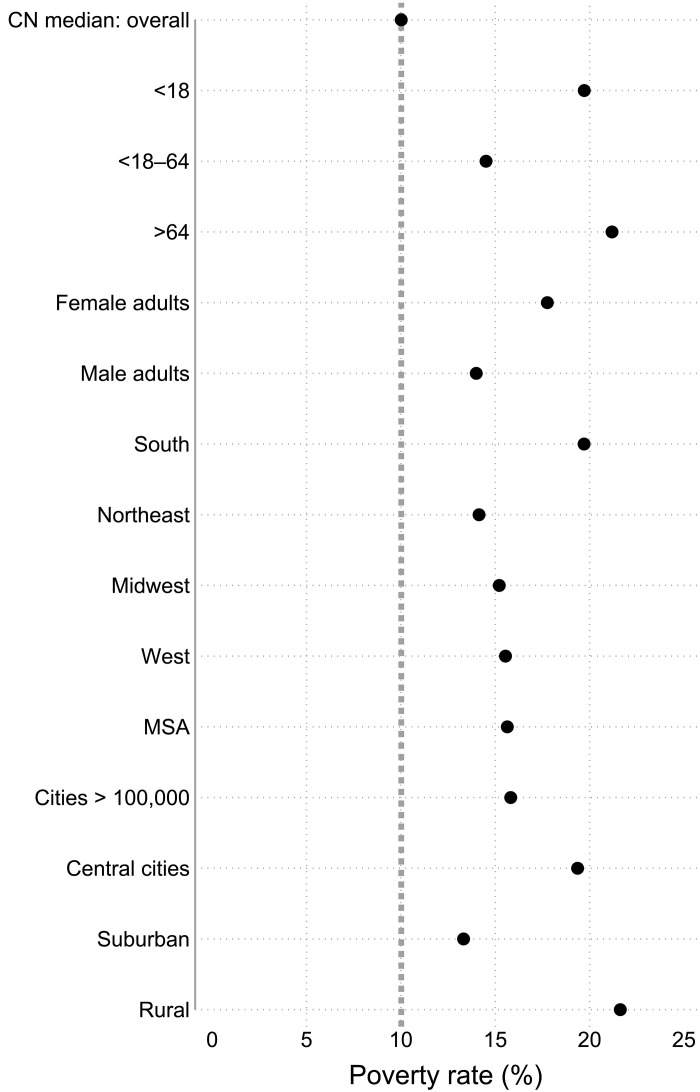
Poverty rates for various groups and places in the United States (2016–2020) and at overall cross-national median. Poverty rates in the United States are much higher than in other rich democracies for a wide variety of groups and places. This is the case for various age groups, adult women and men, and geographical regions and places. The top dot and the vertical dotted line are the cross-national median poverty rate for the overall population. The dots represent poverty rates for various groups and places. Confidence intervals for U.S. estimates are shown as spikes but are too small to be visible (*16*).

U.S. poverty is often viewed as confined to highly disadvantaged risk groups or concentrated inner city poor neighborhoods. The reality, however, is that high poverty is common across groups and places (*38*). Figure 6 shows the poverty rates for several groups and places. Again, these rates are compared against the cross-national median overall poverty rate. Poverty is especially high among those under aged 18 and 65+, in the South, central cities, and rural areas. However, U.S. poverty is also above the cross-national median among working-aged adults (18 to 64), among both adult women and men, in all four regions, and in large cities (i.e. Metropolitan Statistical Areas), moderate cities (>100,000), and the suburbs. Therefore, U.S. poverty is systemically high partly because it is high across groups and places.

## PREVAILING APPROACHES TO THE INDIVIDUAL POOR

Unfortunately, American poverty research has not traditionally aimed to explain the question of the systemically high poverty in the United States. Rather, the prevailing approaches have focused on the individual poor. This partly reflects America’s uniquely strong individualistic ideology (*1*, *4*, *10*, *39*). This section critically reviews three of the prevailing approaches in American poverty research: (i) behavioral explanations that imply “fixing the poor,” (ii) emotive compassion narratives that imply “dramatizing the poor,” and (iii) cultural explanations that imply both dramatizing and fixing the poor.

### Fixing the poor

First, behavioral explanations aim to “fix the poor.” This approach posits counterproductive behavior as the key mechanism causing individuals to be poor. These behaviors are risks or demographic and labor market individual characteristics that are more common among the poor. The four major risks are joblessness, low education, young headship, and single motherhood (*31*). According to behavioralists, the poor are poor because they exhibit a greater “prevalence” (i.e., share of the population) of such risks (*40*–*42*). For behavioralists, the principal anti-poverty strategy is to reduce prevalences of risks (*43*).

In this view, behavior is driven by choices/incentives, culture, and traits: Almost the only causal pathway from choices, culture, and traits to poverty is through behavior. As a result, many concentrate on altering the choices, cultures, and traits of poor individuals. This presumes that disproportionately poor groups have disproportionately problematic or even pathological behavior (*35*). In turn, behavioralists devote considerable effort to scrutinizing and documenting welfare disincentives that lead to problematic behavior (*44*).

According to this literature, poverty also has feedback effects, which reproduce poverty. Researchers argue that poverty imposes a cognitive burden, present bias, and stress, which reduces bandwidth, which then purportedly encourages counterproductive poverty-increasing behavior (*45*–*47*). For instance, Shah and colleagues (*48*) use laboratory experiments to simulate scarcity in games like Wheel of Fortune, Family Feud, and Angry Birds. On the basis of such experiments, they claim that this explains why (p. 682) “The poor often behave in ways that reinforce poverty.”

Public commentary also routinely advances behavioral explanations. Sawhill’s (*49*) “success sequence” is based on her contention that “Those who graduate from high school, wait until marriage to have children, limit the size of their families and work full-time will not be poor.” Academics often offer such behavioralist public commentary as well (*2*). The American Enterprise Institute and Brookings Institution convened two “bi-partisan” “consensus groups” of eminent academics on poverty (*50*, *51*). Both concentrate overwhelmingly on risks and behavior. The 2015 plan emphasizes marriage, delayed parenthood, employment, and education. The plan’s first recommendation is to “promote a new cultural norm surrounding parenthood and marriage.” The 2022 version similarly advocates, “marriage is the best path to favorable outcomes…marriage offers the most reliable way” (p. 22).

Despite being prominent, behavioral explanations cannot explain the systemically high U.S. poverty. This is demonstrable using Brady and colleagues’ (*31*) “prevalences and penalties” (PP) framework, which describes and assesses the risks of poverty (*9*, *52*, *53*). Prevalences are the share of the population with a given risk, which has been the focus of behavioralists. Penalties are the greater probabilities of poverty associated with a risk. Among other topics, the PP framework has been applied to interstate and historical variation in poverty (*54*, *55*), child poverty before and after the Great Recession (*56*), and immigrant child poverty (*57*). Research with the PP framework identifies three critical limitations of behavioral explanations.

First, prevalences cannot explain macrolevel variation in poverty (*58*, *59*). If the prevalences could explain systemically high U.S. poverty, then the United States should have high prevalences of joblessness, low-education, young headship, and single motherhood. However, the United States actually has below average prevalences compared to other rich democracies (*31*). Across 29 rich democracies, the United States has the 20th highest prevalence of at least one risk. The United States is also below average in the two paramount risks: joblessness and low education. Furthermore, despite vast cross-national variation in poverty, there is little cross-national variation in prevalences as all rich democracies have similar prevalences (*31*). As a result, if the United States had cross-national median prevalences for young headship, low education, joblessness, or all four major risks, U.S. poverty would increase, not decline. If prevalences could explain U.S. poverty, then poverty should have declined as a result of the marked historic decline of the risks of low education, joblessness, and young headship (*31*). However, poverty has been stable at a high level since the 1970s. U.S. poverty would be higher if the U.S. returned to historic prevalences of young headship, low education, joblessness, or all four risks.

Behavioralists routinely ask why the poor fail to get married, why they do not complete their education, and why they do not work (or why they do not open bank accounts or why they fail to sign up for welfare programs) (*42*, *47*, *49*). The reality is that the United States actually has cross-nationally below average prevalences and those prevalences have declined considerably over time. Thus, U.S. residents tend to make fewer such choices and engage in fewer such behaviors than in other rich democracies or even the United States historically. Every country has a share of its population making such choices and engaging in such behavior (*60*). Because most Americans play by the rules, the better question is why so few current U.S. residents fail to do so. Because most Americans are not in the risk groups, reducing the size of the risk groups cannot substantially reduce the systemically high U.S. poverty (see Fig. 5).

Second, the causality between behavior and poverty is questionable. The effects of risks are almost always not causally identified, and risks are certainly also endogenous to poverty (*36*). Any coefficient for a risk of poverty is confounded with other risks and other characteristics that are also associated with poverty. All of this artificially inflates the coefficients and exaggerates how much risks matter. Closely related, behavioralists routinely fallaciously imply that “who is poor” explains “why there is poverty” [for critiques of this logic, see (*2*, *5*, *61*)].

The PP framework reveals that risks are actually unreliable predictors of poverty. If risks were reliable predictors, then one would expect that the penalties of risks to be similar over time and across countries. In the past several decades within the United States, however, the association between single motherhood and child poverty has declined substantially (*62*, *63*). Conversely, the association between joblessness and poverty has increased and become more important than single motherhood (*54*, *57*, *62*, *64*). Across rich democracies, the relationships between risks and poverty vary widely and are often quite weak. There is far more cross-national variation in penalties than in prevalences. For instance, in 16 of the 29 rich democracies, single motherhood does not even significantly predict working-aged poverty (*31*). Research shows that the United States is exceptional and atypical for having the highest penalties of any rich democracy (*31*).

This shows how studying only the one country, the United States, where risks are most strongly associated with poverty has biased impressions about the causal relationship between risks and poverty. The United States is not a universal case and is only one of many countries. As Smeeding and colleagues [(*30*), p. 162] point out, the U.S. poverty literature “rests on an inherently parochial foundation, for it is based on the experiences of only one nation.” The United States is an outlier for the dependent variable because of its systemically high poverty and an outlier because of its highest penalties. Generalizing about the relationship between risks and poverty based on an outlier suffers from selection biases just like any sample selection bias (*65*). The researcher does not know what conditions in the outlier U.S. setting are interacting with the risk to augment the penalty (*66*, *67*). The researcher does not know how much poverty is due to the risk itself or highly contingent interactions between the risk and the setting (*6*). Ultimately, any impression about the relationship between risks and poverty based solely on the United States is biased because the United States is an outlier. This problem is exacerbated if one only studies poor neighborhoods or very poor groups in the United States (*6*).

Third, behavioralists mostly focus on prevalences and neglect the more salient penalties. However, there is far more variation in penalties than prevalences (*31*). The United States does not stand out for having high prevalences but does stand out for having the highest penalties among rich democracies. Furthermore, reducing penalties would reduce poverty more than reducing prevalences (*31*). Penalties, therefore, provide a far better explanation of America’s systemically high poverty than prevalences. All of this supports the political explanations below.

### Dramatizing the poor

A second prevailing approach aims to elicit emotion and compassion through humanizing narratives built on ethnography and intensive interviews (*68*–*70*). With granular detail of the daily struggles of the poor (*70*, *71*), this approach offers vivid, often shocking, descriptions of poor individuals (*72*–*74*). The emphasis is often on the desperation and suffering of extremely poor individuals stuck in impossible situations, often in places of concentrated extreme poverty (*68*, *75*). Books in this approach routinely have evocative titles like *On the Run*, *$2.00 A Day*, *Living the Drama*, *American Dream*, and *Gang Leader for a Day*.

Desmond and Western (*69*) argue that this “new direction” emphasizes “poverty is morally urgent…an affront to dignity.” Desmond and Western even go so far as to say, “Esteeming dignity encourages a humanizing social analysis, where researchers are sensitized to the capacity for love, creativity, and imagination in their subjects. The principle of human dignity also *shifts the poverty debate away from income redistribution*” (emphasis added). This is noteworthy given that political explanations discussed below tend to emphasize redistribution as necessary and perhaps more salient than almost any other cause of variation in poverty (*76*).

Compared to behavioralists, this approach has clear advantages. It reveals the exceptionally constrained and near impossible “choices” that poor people have to make, which humanizes the poor. Such narratives of the poor’s actual daily lives (*70*) are surely more construct valid than laboratory experiments using video games (*48*) or textbook assumptions about welfare disincentives. This approach also implies an intuitive theory about causes and solutions. By humanizing and dignifying the poor, this literature uses narratives to get readers to become sympathetic. Such sympathy is then expected to set the agenda for anti-poverty policies (e.g., shifting politicians away from punitiveness). This implies that a lack of humanization is a critical cause of why the United States has systematically high poverty. Below, I revisit whether this is a convincing political theory of poverty.

No matter the intent to humanize, this approach still ends up focused on why certain dramatized individuals are poor—their life history, unfortunate circumstances, or counterproductive behavior that caused such dire straits—rather than why the United States has systemically high poverty. To paraphrase Matt Bruenig, one cannot simply “collect a bunch of personal stories and then zoom out” to an effective theory of poverty. It does not follow that compassion or moral urgency clarifies what causes poverty or how we should intervene on it. Many have tremendous compassion for the poor but maintain an individualistic theory of the causes of and solutions for poverty. Dramatizing individual poor people may even obscure that the actual cause of the plight of these individuals is the systemically high poverty. Concretely, there are three main limitations to dramatizing the poor.

First, this approach overemphasizes the unrepresentative groups of the poor. It is fair to view texts as representations of the poor, and one should scrutinize whether texts accurately represent those in poverty (*2*, *77*, *78*). Unfortunately, this literature disproportionately represents the poor as visible, stigmatized, minoritized, and perhaps “exotic” groups. For instance, unlike the heterogeneous recipients of Aid to Families with Dependent Children, DeParle (*73*) highlights three unrepresentative young Black single mothers with high fertility and multiple partners, who move from Chicago to Milwaukee for more generous welfare benefits and become addicted to crack cocaine. Contrary to the representation by Goffman (*74*), the overwhelming majority of those in poverty are not Black adolescents with outstanding warrants. Contrary to Bourgois (*72*) and one of the eight cases by Desmond (*68*), the overwhelming majority of those in poverty do not sell drugs. Contrary to the one of the eight of cases by Desmond (*68*), most people in poverty do not spend nearly their entire welfare benefits on a lobster dinner. This approach may lean on the theory that humanizing narratives will evoke compassion for such groups. However, mischaracterizing the population in poverty and overrepresenting exotic subgroups also fuels unflattering stereotypes about the poor. Such unflattering misrepresentations are likely to backfire and have even larger adverse political consequences (*2*, *77*, *78*).

**Fig. 7.**
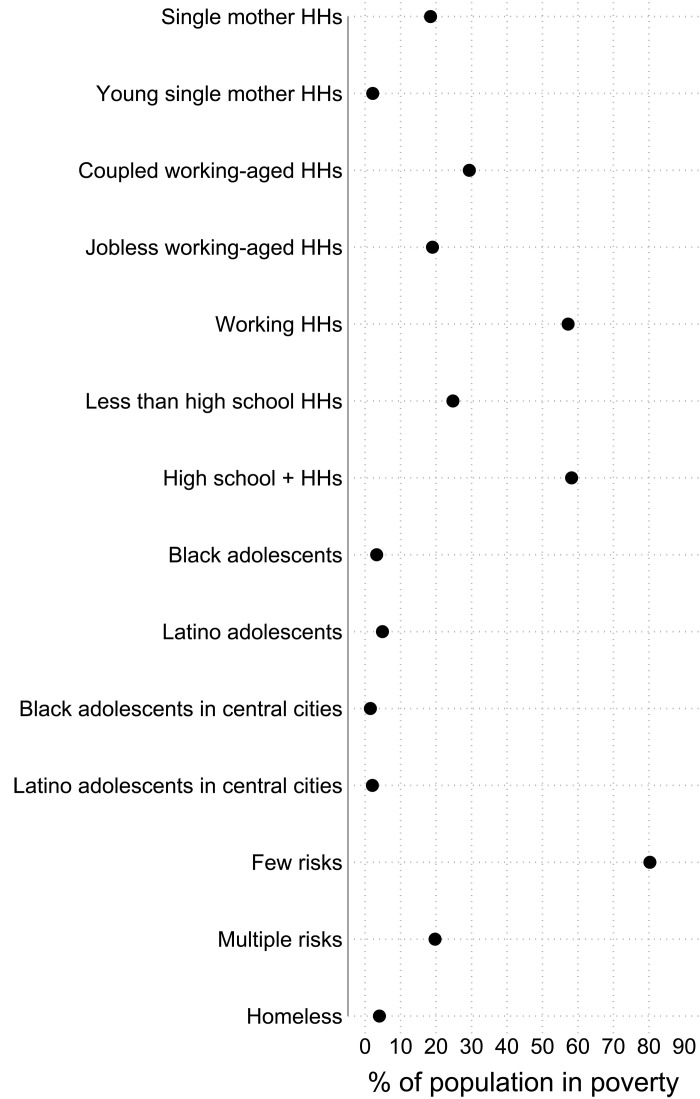
Stereotypical versus typical shares of the percent of the population in poverty in the United States (2016–2020). Stereotypical groups actually represent a small share of the U.S. population in poverty. The typical groups in poverty actually exhibit few risks and are a much larger share of the U.S. poverty population. The dots represent the share of the U.S. poverty population. Confidence intervals for U.S. estimates are shown as spikes but are too small to be visible (*16*).

Figure 7 reveals that such stereotypical groups are actually a small share of the population in poverty. Among those in poverty, the share in single mother households (18.5%) is much smaller than the share in coupled working-aged households (29.4%). Only about 2.1% of poor people are in young single mother households. Among those in poverty, about three times more are in employed than jobless households (57.2% versus 19%). There are also more than two times as many poor people in households led by someone with at least a high school degree than someone without (58.2% versus 24.8%). Ethno-racial minority adolescents are perhaps the most dramatized poor. However, Black and Latino adolescents amount to only 3 and 5.5% of people in poverty. Black and Latino adolescents in the highly studied inner cities are merely 1.4 and 2.3% of people in poverty. Far fewer are in poor neighborhoods. People with multiple risks (e.g., low educated, jobless, and young single mothers) are frequent in this literature. However, there are more than four times as many poor people with zero or one risk versus those who have 2+ risks (80.3% versus 19.7%). Last, a crude estimate is that about 4% of the population in poverty is homeless. This estimate uses the national point in time count for 2019 and follows Evans and colleagues [(*79*), p. 924], “the point-in-time estimate understates annual exposure by a factor of almost four.”

The reality is that people in poverty look similar to the United States as a whole. If one were to construct a composite “typical” poor person in the United States based on the most common populations in multiple categories, it would be a white, 35- to 45-year-old woman, with a high school education, in a coupled and employed household with no children, and living in a metropolitan area in the South. It is important to stress that acknowledging the reality of the population in poverty should not downplay that, as Figs. 2 and 3 show, Black and Latino children have staggeringly high poverty. However, concentrating so disproportionately on such groups misrepresents the typical people in poverty.

Figure 7 also reveals how dramatizing the poor provides a weak basis for reducing poverty. Even if these stereotypical risk groups have a high probability of poverty, that does not mean reducing the size of these groups will substantially reduce overall poverty. Because these stereotypical risk groups are unexpectedly small, poverty would remain systemically high even if such risks were eradicated. For instance, Native American child poverty is staggeringly high. However, even if Native American child poverty was eliminated completely, the United States would maintain systemically high poverty. To reduce the systemically high U.S. poverty, we need to understand not only who is more likely to be poor but also who is in poverty.

Second, dramatizing the poor disproportionately focuses on symptoms rather than underlying causes. The clearest example is the enormous attention on eviction. Recall, Fig. 1 shows that almost 64× as many people were in poverty as were evicted in 2019. For this reason alone, eviction is simply not one of the more salient causes of poverty. In addition, even if eviction has a salient causal effect reproducing or exacerbating poverty (and such evidence has been thin), eviction is far more a symptom than a cause. If incomes were higher, the threat of eviction would be lower. Therefore, the more important question is why so many have low incomes and why there is systemically high poverty, not why people with low incomes are more likely to be evicted. It seems reasonable to question whether the enormous attention on eviction or other symptoms crowd out attention to the more salient and underlying causes of poverty (see political explanations below).

Third, dramatizing the poor ends up downplaying effective social policies. Because this literature prioritizes eliciting compassion about the suffering of the extremely poor, it has an incentive to make poverty seem as bad and evocative as possible. This is partly accomplished by discounting evidence that social policies are reducing poverty. Unfortunately, this fuels a false impression of futility that social policies do not reduce poverty, what can be called the fallacy of intractability. By accurately incorporating social policies, we more accurately characterize the population in poverty and accurately reveal that social policies are effective.

Edin and Shaefer’s (*75*) celebrated *$2.00 A Day* measures extreme poverty at the remarkably low threshold of $730 a year. Edin and Shaefer claim that there was a marked growth in $2/day poverty; the growth was exacerbated by the 1996 welfare reform; the growth was especially worse among single mothers with children; and there are notably high numbers with less than $2 per day. The problem is that they measure household income as only cash income and omit some welfare transfers as well as taxes and tax credits. In particular, they omit SNAP, which is actually a critically effective social policy for extreme poverty. By contrast, Brady and Parolin (*11*) set a more reasonable threshold and measure income comprehensively. They show that Edin and Shaefer mischaracterize the rates of and trends and population in extreme poverty. Most people in extreme poverty are childless adults, and SNAP substantially reduces extreme poverty for single mothers with children. While children are actually underrepresented among the extreme poor, Edin and Shaefer even mischaracterize which children are extremely poor (*80*). Rather than children in single mother households, the biggest group is in households headed by immigrants. The reason for this is that immigrant households have far greater difficulty accessing SNAP, which, again, is the critical social policy for reducing extreme poverty.

### Cultural explanations

Cultural explanations are one of the most prominent behavioral theories. Here, I am referring specifically to the argument that a problematic or pathological culture causes counterproductive behavior and risks that cause poverty. Furthermore, poverty feeds back into and reinforces that problematic culture, often intergenerationally (*81*). Like fixing the poor, culturalists require behavior as the mechanism between culture and poverty. Like dramatizing the poor, culturalists highlight the granular daily experiences and meanings of the poor.

Cultural explanations have been around seemingly forever (*1*, *4*). After the 1960s, however, cultural theories justly received tremendous criticism for flawed generalizations about poor people, ethno-racial minorities, and developing countries (*6*, *82*, *83*). Some attribute the subsequent decline of cultural explanations to political controversies around them (e.g., the Moynihan Report) especially in the 1970s (*35*). However, such a partisan narrative elides and minimizes that cultural explanations suffered from substantial logical and evidentiary problems. Those problems are a far more plausible source of its downfall (*1*, *2*, *4*, *6*, *84*).

Cultural explanations were always hopelessly endogenous: If culture caused poverty and poverty caused culture, then what causes the systemically high poverty in the first place? Cultural explanations usually suffered from sample selection biases, as they concentrated on poor people or poor neighborhoods without comparison groups. Cultural explanations often stereotyped the deviant or pathological values and behaviors of disadvantaged groups. Worse, those stereotypes were often based on the subjective biases of elite white male academics. Ample evidence has long shown that it is false that the poor have anti-marriage (*83*, *85*) or anti-school values (*86*). Culturalists almost never compared their account against rival theories. Scholars rarely showed evidence that culture mattered net of other causes or how the magnitude of any cultural effects compared against other causes. Rather than adolescent Black males lacking soft skills (*87*), ample evidence shows extensive racial discrimination by employers. Rather than the poor being ignorant about college pathways (*71*), highly segregated and lower quality schools and toxic environments likely undermine educational attainment (*36*). It is possible that culture does not matter net of such factors, which plausibly have even larger effects.

Moreover, culturalists never really even attempted to explain systemically high poverty. By narrowly focusing on some selectively chosen specific poor group, cultural explanations notoriously routinely neglected the political causes discussed below. Indeed, cultural explanations habitually omitted the historical and institutional contexts underlying systemically high poverty. For the most part and for many, such problems and critiques were persuasive (*1*, *2*, *4*, *6*, *83*, *84*).

Hence, it was surprising that, in 2010, the *New York Times* reported, “‘Culture of Poverty’ Makes a Comeback” (*88*). Highly publicized Harvard University sociologists were advocating for cultural explanations of poverty again. Even more surprising, these “new culturalists” did not really address the critiques of prior cultural explanations. Instead, a narrative was constructed about the courage of these Harvard sociologists in overcoming political correctness in being willing to touch what Harding [(*71*), p. 5] called the “third rail of scholarship on urban poverty.” Massey claims, “We’ve finally reached the stage where people aren’t afraid of being politically incorrect” (*88*). Small refers to themselves as a “new generation of scholars without the baggage of that debate” (*88*). Their 2010 *Annals* issue “Reconsidering culture and poverty” has been among the most highly cited pieces of poverty research in recent decades.

The new culturalists adopt rhetorical strategies to distinguish their approach from older cultural explanations (*89*). They use euphemistic vocabulary of scripts, schema, and frames rather than the older values and norms (*90*). They stress that culture is not homogenous, pathological, or deviant but heterogeneous and adaptive (*71*). They also stress that culture has a probabilistic rather than deterministic relationship with poverty.

Nevertheless, the new culturalists ultimately recreate the same argument that problematic culture causes problematic behavior, which causes poverty (*81*, *89*). I add italics to substantiate this point. Small and colleagues’ [(*90*), p. 6] aim is “explicitly *explaining the behavior* of low-income population in reference to cultural factors” and demonstrating “processes and mechanisms that lead to the *reproduction of poverty*” (p. 23). Small and colleagues [(*90*), p. 15] write, “Rather than causing behavior, *frames make it possible or likely*.” However, there really is no difference between “cause” and “make possible or likely.” Small and colleagues [(*90*), p. 23] further claim culture, “should become central to our understanding of the *production and reproduction of poverty and social inequality*.” Harding [(*71*), p. 14] writes, “Variation in repertoires…will lead to divergent behaviors, and it is in this way that *culture plays a causal role in influencing action*.” Vaisey [(*91*), p. 96] argues culture “might have an ‘*exogenous explanatory power’ that serves to inhibit socioeconomic success*.”

In one of the most influential new culturalist studies, Harding (*71*) argues that cultural heterogeneity causes poverty-increasing behavior. Harding interviews 60 Black and Latino adolescent boys and conducts “surgical fieldwork” (p. 14) in three mainly poor Black neighborhoods in Boston. While discounting that his race, sex, and class constrain interviewing or interpretation (pp. 16, 20, 265), Harding (p. xii) dismisses political explanations that imply “victims of structural forces beyond their control” and derides implying “individuals floating like feathers buffeted by winds produced by economic and social forces beyond their control.”

Harding argues “model shifting” between and “simultaneity” of good and bad, and “dilution” of good cultural scripts, frames, and schemas cause boys to not use condoms and resist commitment to girls, engage in violence, and “produce ignorance about how to successfully navigate educational institutions” (p. 226). While disavowing homogenous values and norms, the argument ends up in the same place as older cultural arguments. “Ghetto-specific cultural models” (pp. 6, 243), “negative role models” (pp. 67, 241), and “level expectations” (pp. 57, 61). The text mainly concentrates on mean differences in culture/behavior of disadvantaged Black/Latino boys/neighborhoods versus unobserved non-disadvantaged boys and neighborhoods. In poor neighborhoods, “cross-cohort socialization” (p. 72) exposes boys to “ways of thinking about problems, solutions, and decisions that are sometimes at odds with mainstream or middle class convention” (p. 91), which “affects adolescent decision making and behavior” (p. 104). Similarly, “Adolescent boys who have little or no relationship with their fathers, the norm in poor neighborhoods, are particularly susceptible to the influence of older peers” (p. 104). Ultimately, “Heterogeneity in cultural lifestyles and orientations can be understood as the failure of more middle-class or mainstream-oriented residents of disadvantaged neighborhoods to control behavior in their communities” (p. 244).

Despite euphemistic vocabulary and sidestepping prior critiques, the new culturalists face the same problems as the old (*83*, *84*): endogeneity, selection biases, subjective biases, lack of contrast against rival explanations, lack of comparison groups, etc. Ultimately, the new culturalists compound the problems of both fixing and dramatizing the poor. Like fixing the poor, the new culturalists cannot explain the systemically high poverty that sets the stage for their studies. For instance, they cannot explain what fundamentally causes the staggeringly high poverty of Black and Latino adolescent boys in concentrated poor neighborhoods. In addition, the new culturalists do not address that the problematic behaviors they emphasize (e.g., single motherhood) are often unreliable predictors of poverty. They also do not address that the penalties attached to such behaviors can be politically moderated. Nor do they acknowledge that penalties are lower in every other rich democracy except the United States. Like dramatizing the poor, the new culturalists fixate on symptoms more than underlying causes. The new culturalists also overrepresent stereotypical poor groups and misrepresent the population in poverty.

## POLITICAL EXPLANATIONS OF SYSTEMICALLY HIGH POVERTY

According to political explanations, power, policies, and institutions are the pivotal cause of poverty (*10*, *76*, *92*). Indirectly, power and institutions cause policy, which causes poverty. Power and institutions also reinforce each other. Rather than presuming that risks cause poverty like behavioralists, political explanations emphasize that policy and institutions moderate the relationship between risks and poverty. Contrary to prevailing approaches, political explanations provide a far better account for why the United States has systemically high poverty. Political explanations see individuals as poor largely because of that systemically high poverty.

While the three prior approaches have traditionally prevailed (*3*, *4*), political explanations have risen in prominence in recent decades. Before roughly 2000, there were few studies that would be explicitly labeled as political explanations. Partly, this rise occurred because of marked advances in cross-national income data and especially the LIS database (*7*). Such data enabled scrutiny of taxes and transfers as well as variations in policies and institutions. If a researcher only ever studies one political context, then the horizons of explanation are constrained because of selection bias. If one only studies the United States, without comparison to other countries, then this leads to a sample selection bias where one cannot answer why the United States has comparatively high poverty. To paraphrase Lipset (*93*), poverty scholars who only know one country, know no country.

Political explanations recognize that poverty normally occurs in a context of sufficient or even abundant resources rather than scarcity. Societies usually have sufficient resources that, if they were distributed more equally, then poverty could be reduced. Even during famines, as Sen (*94*) famously explained, resources are available nearby. This is why famines are almost always prevented in well-functioning democracies. Similarly, in rich democracies, there are abundant resources. The problem is that resources are not well-distributed, and poverty, therefore, is due to distribution not scarcity (*95*). Because distributions are almost always shaped by politics, poverty is actually a political problem (*3*, *10*, *92*). The political problem is more consequential than how the poor behave in response to some artificially imposed scarcity [cf. (*48*, *71*)].

Political factors are often included within “structural” explanations (*4*, *5*, *95*–*99*). Both structural and “political” explanations shift away from individual risks to contextual causes of poverty. However, I draw a meaningful distinction between structural explanations (emphasizing demographic and labor market contexts) and political explanations (emphasizing policies, power and institutions). It may be best to label structural explanations as emphasizing demographic and labor market contexts (e.g., deindustrialization) and not vacuously using “structure” as a catchall for every social force above an individual. It may then be best to label political explanations as emphasizing policies, power, and institutions. This distinction clarifies that institutions and policies more strictly reflect politics than demographic and labor market contexts. As Brady [(*76*), p. 168] remarks, “While structuralists view poverty as the unfortunate byproduct of contextual factors that overwhelm what can be done, political accounts view poverty as the willfully chosen outcome of state (in)action when something could be done.”

In his genius metaphor, Rank (*5*) illustrates his structural vulnerability theory with a game of musical chairs. A scarcity of chairs (e.g., good jobs), not the individuals’ quickness at seizing a chair (i.e., behavior), explains why someone is left without a chair (i.e., poor). A political explanation would instead say that there is not so much a scarcity as a distribution problem. Rather than too few chairs, one person is laying across and hoarding three, several chairs are disrepaired and piled nearby in the corner, and someone is then told that they have no place to sit because of what is disingenuously framed as a scarcity.

Within political explanations, there have been at least four concrete themes. I now elaborate on each.

### The essential role of social policy generosity

The strongest and most robust generalization to emerge from recent decades of political research on poverty is that social policy generosity is a requirement for low poverty. At the risk of hyperbole, there has never been a country in the modern history of capitalist democracies that accomplished sustainably low poverty without a large welfare state. Consistently, across a variety of samples of countries, years, and states, there is a clear, strong, and robust negative relationship between the welfare state and poverty (*10*, *29*, *92*, *100*–*111*). The powerful role of social policy was made very clear by how U.S. poverty declined during COVID. Because the U.S. government extended unemployment insurance, expanded the CTC, and provided stimulus and relief payments, poverty was considerably lower (*14*).

Generous social policies reduce poverty regardless of whether one measures social policy generosity as formally legislated social rights (*112*, *113*), as the amount of welfare transfers actually received (*102*, *107*, *114*), or as the totality of welfare spending (*10*, *105*). “Generosity” is shorthand for the combination of ways equality-enhancing social policies are more universal and have high replacement rates, lower eligibility thresholds, and high coverage (*112*, *113*). Generosity also often refers to constellations of dimensions of welfare programs, such as universalism and high levels of “transfer share” (*102*).

In a compelling recent example, Alper and colleagues (*115*) show that both social rights and welfare spending enhance poverty reduction and reduce poverty after taxes and transfer. Social policies reduce poverty through taxes and transfers and publicly provided services. Innovative research shows that, if we monetize publicly provided health insurance, then the expansion of Medicaid under Obamacare substantially reduced poverty (*116*, *117*) and economic insecurity (*118*). Social policy generosity is also the product of both the legislation and implementation of such policies. In turn, the execution of social policy and the administrative burdens imposed on potential recipients of social policies clearly influence how generous social policies actually are on the ground (*78*, *114*, *119*–*121*). Closely related, racial disparities in implementation and discrimination in access to generous social policies are key drivers of racial inequalities in poverty (*32*, *122*, *123*).

These patterns go far beyond the internal validity of identifying the local causal effect of a specific social policy (*44*, *100*, *124*, *125*). The literature has built an externally valid generalization that generous welfare states have lower poverty. The evidence has accumulated across a wide variety of cross-national as well as within-nation studies, and the pattern has cemented especially as cross-national income and poverty data have matured. Particularly important for this review, social policy generosity is, presently, the best explanation for why America has systemically high poverty (*10*).

**Fig. 8.**
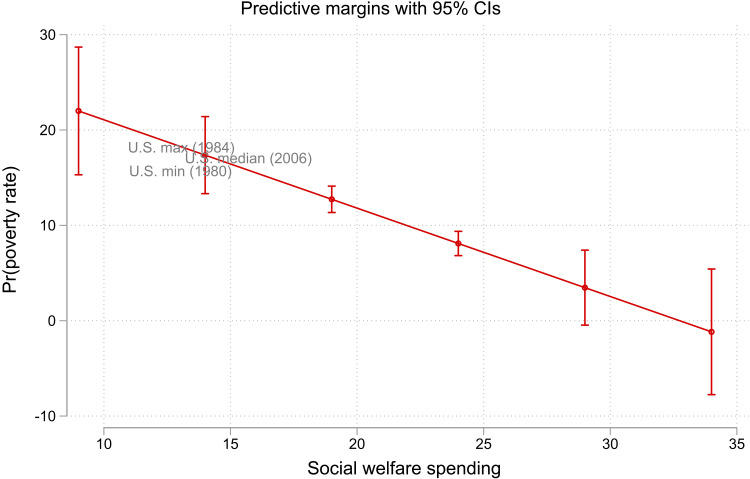
Predicted poverty rates from model of social welfare spending in 20 rich democracies (1980–2019) overlayed with actual U.S. minimum (1980), U.S. median (2006), and U.S. maximum (1984) poverty rates. There is a strong and significant negative relationship between poverty rates and social welfare spending. The actual U.S. poverty rates are very well predicted by the fact that the United States has low levels of social welfare spending. The solid downward-sloping line is the predicted poverty rates [95% confidence intervals (CIs) are shown as error bars]. The estimates are based on a two-way fixed-effects model (country and year) of poverty rates on social welfare spending and its square (as a percent of gross domestic product).

As an illustration, Fig. 8 replicates prior analyses of poverty across rich democracies (*10*, *105*, *115*). Figure 8 shows results from a fixed-effects (for country and year) model of all 20 rich democracies with data in the OECD and LIS from 1980 to 2019. The predicted values in Fig. 8 show that social welfare spending as a percent of gross domestic product has a significant negative relationship with poverty rates. As is by now well-established, as social policy generosity increases, poverty declines.

Figure 8 also overlays markers for the actual U.S. minimum, median, and maximum poverty rates. This shows that the high actual poverty rates of the United States fall almost exactly into line with the model’s predicted poverty rates. The United States has systemically high poverty because it has low social welfare spending. The United States is always in the upper left part of the figure. This confirms the United States is a perennial outlier, both in terms of high poverty and low social welfare spending. This demonstrates that one will misunderstand poverty if one only studies the United States.

### Political choices to penalize risks

Behavioralists and culturalists contend that individual counterproductive choices explain why some individuals are poor and other individuals are not. By contrast, political explanations contend that states make political choices about which risks will be protected and which will not. By “political choices,” I mean a realistically nuanced “decision” by a polity to act or not. Sometimes, politicians do deliberate in proverbial smoke-filled rooms for policy that worsens poverty (e.g., a few Senators’ choice to not continue President Biden’s expanded CTC or state legislatures’ choice to not expand Medicaid). Of course, political choices also include public opinion, agenda setting, and other processes. The point is that policies and penalties are not inevitable, and not acting is a choice just like acting is a choice.

According to the PP framework, countries politically decide how much to penalize risk groups. Behavioralists and culturalists presume a mechanical, automatic link between individual risks and individual poverty and, therefore, emphasize reducing the prevalences of risks. By contrast, political explanations stress that the relationships between individual risks and poverty vary tremendously across political contexts. This variation in penalties is politically manipulable. Therefore, political explanations emphasize reducing penalties (*31*).

As mentioned above, that penalties vary more and matter more to poverty has now been demonstrated by research across rich democracies and the United States over time (*126*). For instance, single motherhood has been arguably been the most well-studied risk by behavioralists and culturalists. Across 29 rich democracies, Brady and colleagues (*31*) find more than three times as much variation in single motherhood penalties as prevalences. Similarly, Nicholson (*54*) finds that, while interstate variation in single motherhood prevalences was stable 1993–2016, interstate variation in single motherhood penalties more than doubled. Moreover, the United States does not stand out for having a high prevalence of single motherhood. Rather what makes the United States stand out is having the highest penalty for single motherhood (*127*, *128*).

Such variation and, especially, the extremely high penalties for single motherhood and other risks in the United States have been convincingly linked to politics. The United States politically chooses to penalize single mothers and other risk groups far more than other rich democracies. Variation in social policy generosity moderates the single motherhood penalty for child poverty and for poverty among families with children (*128*). Variation in social policy also moderates the penalties attached to the more salient risks of low education and joblessness (*31*). Because penalties matter more to poverty than prevalences, social policy is a critical factor explaining the relationships between risks and poverty. Moreover, that the United States has such exceptionally high penalties reinforces how unusual the U.S. case is and how studying only the United States biases conclusions about poverty.

### Power resources of collective political actors

Underlying and causing generous social policies, collective political actors mobilize less advantaged classes into “power resources” (*10*, *103*, *108*, *129*–*131*). According to power resource theory, labor unions and leftist parties unite workers and voters around shared interests and ideology to push for generous social policies (*37*, *132*, *133*). The mobilization of the less advantaged is pivotal because the default distribution of political power in a capitalist democracy favors elites and business (*134*). Hence, it is essential for the working class and poor to bond together and attract some of the middle class to gain any real political power. Power resource theory thus provides a theory of the income distribution, with the welfare state as a principal mechanism and collective political actors as the underlying fundamental cause (*10*, *108*, *129*).

Poverty is lower where and when Left parties have controlled government, unionization is higher, and women are a greater share of parliaments (*10*). Countries with higher unionization have significantly lower working poverty and lower poverty overall (*133*, *135*–*140*). For example, Pineda-Hernández and colleagues (*141*) analyze 24 developed countries from 1990 to 2015. They demonstrate that centralized collective bargaining systems, greater bargaining coverage, and higher unionization significantly reduce working-age poverty after taxes and transfers. They conclude that this is mostly because of the political strength of these power resources in promoting more generous social policies.

This pattern holds across U.S. states as well (*37*, *101*, *140*). Using the PSID 1976–2015, VanHeuvelen and Brady (*142*) provide the first individual-level longitudinal analysis of household-level union membership and state-level unionization and poverty. Both union membership and state unionization have statistically and substantively significant negative relationships with relative and anchored working and working-aged poverty. Household union membership and state unionization significantly negatively interact, augmenting the poverty-reducing effects of each. Higher state unionization also spills over to reduce poverty among nonunion households. These powerful effects of unions are notable because, as VanHeuvelen and Brady write, “American poverty research largely neglects labor unions.” Compared to the enormous attention on eviction for example, I conjecture that low unionization is a more salient cause of poverty.

The focus on political actors inverts the traditional emphasis. Rather than yet another study of the choices of the individual poor, the field needs more study of the choices of political actors who have power over the poor (*10*, *131*, *143*–*146*). Rather than the marked and evocative experiences or culture of exaggerated unrepresentative poor groups, the field needs more study of the routine and banal ways in which powerful actors exclude those in poverty. Whether collective political organizations, individual policy-makers, or street-level bureaucrats, the poverty literature needs more attention to how political actors drive the amount of poverty in society (*2*, *10*, *95*). For example, the poverty governance literature demonstrates how health care workers (*146*), police (*144*, *147*), welfare case workers (*78*, *120*), and local community leaders (*145*) regulate the poor (*134*).

A focus on political actors also invites skepticism about the political theory implicit in dramatizing the poor. Even if humanization triggers some sympathy, it seems unrealistic that sympathy will displace the ideologies and interests that normally motivate political actors. Even if humanization inspires those already sympathetic, getting political adversaries to read such narratives seems unlikely. On some level, political actors reflect public opinion. However, while there is no evidence that the United States has comparatively low levels of compassion, vast evidence shows the American public has a uniquely strong individualistic ideology (*1*, *4*, *10*, *39*). Moreover, it has never really been proven that poverty scholarship—dramatizing, humanizing, or otherwise—actually has much influence on policy-makers or public opinion.

### Institutions

In addition to the mobilization of collective political actors, the politics of poverty is a function of institutions such as laws and regulations (*106*, *108*). Consistent with U.S. poverty being stable at a high level, inequalities tend to be slow-moving and do not respond promptly to electoral changes. There is path dependency to poverty, and, to understand the institutional sources, we need a long-time horizon of causes and effects. Institutions reflect the residue of the power of collective actors in the past, and institutions remain consequential even without active maintenance by collective actors (*103*, *108*). A strong version of institutionalism would claim that established institutions dominate over contemporary politics to lock in poverty. A weaker version of institutionalism would claim that established institutions guide how and when politics can shape poverty.

Some of the classic institutions that have been convincingly linked to poverty include slavery (*148*, *149*), federalism (*38*, *150*), electoral systems (*10*, *103*), democratization (*132*), colonialism (*151*), and criminal justice (*144*, *152*). One extensive literature is on labor market institutions (*105*). Poverty is significantly lower where wage bargaining is coordinated, centralized, and corporatist, and employment contracts are protected (*133*, *135*–*138*, *153*). There is also evidence that poverty is lower where minimum wages are regulated at a higher level (*154*–*156*). Recently, there has been growing interest in how institutional variation in eviction and housing laws shape poverty [e.g., (*157*)].

Institutions in terms of citizenship laws and rules greatly influence immigrant poverty (*57*, *158*). Baker and colleagues (*9*) find that nativity and citizenship explain the largest share of Latino-white and Asian-white poverty gaps. Baker and colleagues demonstrate that high immigrant poverty is not attributable to behavior. Rather, citizenship laws and their enforcement prevent access to social policies (*80*, *158*), constrain employment rights and labor market opportunities (*119*), and limit residential opportunities and decisions (*159*). As a result of these institutional processes and not because of behavior, poverty among immigrants is quite high in the United States. Immigrants could be the most understudied population in poverty in the United States. Recall, it is actually children of immigrants, not single mothers, who are most vulnerable to extreme poverty (*80*).

**Fig. 9.**
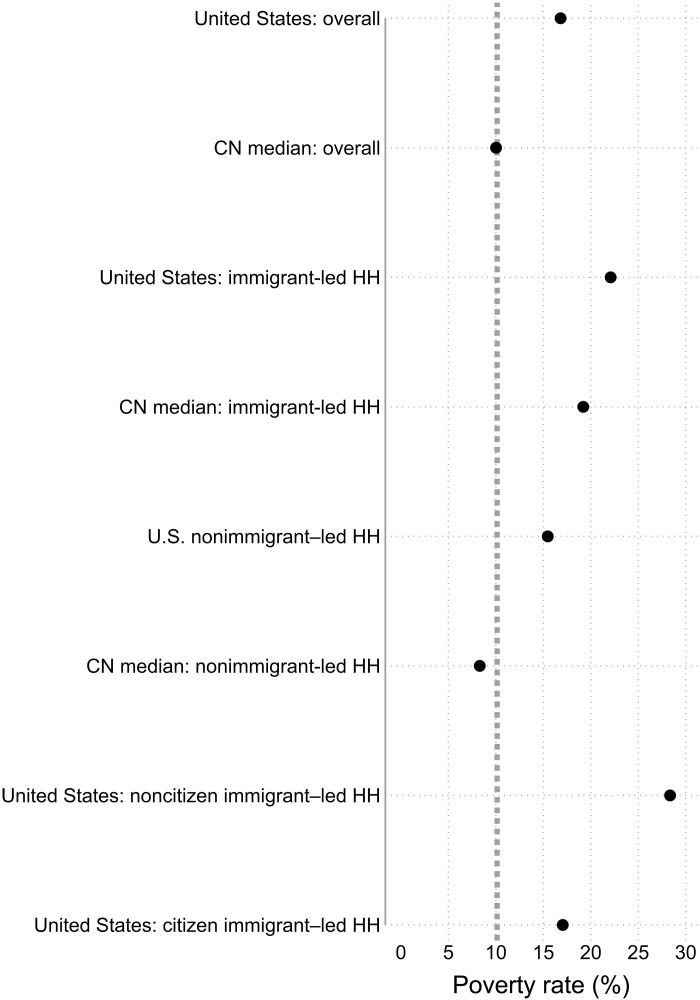
Poverty rates by immigrant and citizenship of household in the United States (2016–2020) and at cross-national median for 24 rich democracies. Poverty rates for immigrant-led households in the United States are higher than poverty rates for the overall population and for households led by nonimmigrants. Across rich democracies, immigrant-led households have higher poverty rates than nonimmigrant households. U.S. noncitizen immigrant–led households have much higher poverty rates than all these groups. CN median is the cross-national median across 24 rich democracies. The vertical dotted line is the cross-national median poverty rate for the overall population. The dots represent poverty rates for various groups. Confidence intervals for U.S. estimates are shown as spikes but are too small to be visible (*16*).

Figure 9 displays poverty rates by immigration characteristics (again, benchmarked with overall poverty). Immigrant-led households in the United States have a poverty rate of 22.1%. This is high compared to poverty among the overall population and among nonimmigrant households. This is also high compared to immigrant households in other rich democracies. Compared to the overall U.S. population, citizen immigrant households actually have similar poverty. What really stands out is the notably high 28.4% poverty rate of noncitizen immigrant households.

There is convincing evidence that institutions drive the staggeringly high poverty of ethno-racial minorities (*99*, *150*). Perhaps the canonical literature is on state-supported segregation (*160*, *161*). Massey and Denton (*34*), for instance, show how federal, state, and local governments and the federalism embodied in all three using or withholding power made countless political choices to institutionalize residential and school segregation. States facilitated (or even subsidized) segregation and under-enforced discrimination law. All of this concentrated poverty, undermined mobility out of poverty, and exacerbated the interaction of race and poverty. Whereas residential preferences of individual whites (and others) are relevant, the state’s role in institutionalizing segregation and discrimination demonstrates political processes driving poverty (*162*).

In a compelling demonstration of how powerful institutions are, Baker (*163*) shows that racial inequalities in poverty in the U.S. South are influenced by historical racial regimes (HRRs). HRRs capture myriad state-level historical racist institutions of slavery, sharecropping, politicians’ opposition to integration, and disenfranchisement devices. Southern states with a higher HRR score tend to have higher poverty. However, this pattern is much more pronounced for racial inequalities in poverty. While white poverty is uncorrelated with HRR, Black poverty and racial inequalities in poverty are strongly positively associated with a state’s HRR. Using multilevel models, Baker demonstrates that HRR exacerbates Black disadvantages in poverty. Using decompositions, Baker shows that HRR explains much of the Black-white poverty gap.

As is probably clear by now but should be underlined, institutions provides a clear path to emphasize racism in theories of poverty. Political explanations in general and institutions in particular explicitly embrace that systemically high poverty, especially the staggeringly high poverty of ethno-racial minorities, is driven by racism. This contrasts sharply with the three prevailing approaches, which rarely explicitly incorporate racism. For example, Small *et al.* (*90*) have only one mention of “racism” (and zero of discrimination), and it regards how young men perceive racism affected them. Harding [(*71*), p. 19] has only one index entry for racism, and it is where he dismisses structural explanations: “social forces far beyond their control – racism…”. Contrasted with classics like Massey and Denton (*34*) or Ryan (*6*), the field appears to have shifted away from racism. Relatedly, new culturalists have a habit of dismissively putting “blaming the victim” in pejorative quotes as if it was not the serious critique it is.

Political explanations reject blaming the staggeringly high poverty of ethno-racial minorities on their behavior or culture. Rather, political explanations emphasize that historical and present state policies—such as slavery, federalism, electoral systems, colonialism, criminal justice, and segregation—are pivotal to racial inequalities in poverty (*3*, *9*, *123*, *148*, *163*, *164*). Racism obviously also exists in and drives variation in social policy (*32*, *78*, *122*), the penalties for risks (*33*), and the power of collective political actors (*101*). Racism within all of these political factors provides a more persuasive account of racial inequalities in poverty than behavior or culture.

## CONCLUSIONS

To fully understand poverty in the United States, the field should center attention on the systemically high poverty. U.S. poverty affects a huge share of the population, is a perennial outlier among rich democracies, is staggeringly high for certain groups, is unexpectedly high for those who play by the rules, and is pervasive across various groups and places. Unfortunately, American poverty research has traditionally not prioritized the understanding of the systemically high U.S. poverty and has instead concentrated on poor individuals. American poverty research has focused on the problem of persons and the poor, not poverty. The field has devoted disproportionate attention to behavioral explanations fixing the poor, emotive compassion dramatizing the poor, and cultural explanations both dramatizing and fixing the poor. This review argues that prevailing approaches cannot explain the systemically high poverty in the United States. Partly as a result, prevailing approaches are unlikely to provide effective policies to address the causes of poverty.

Instead, this review advocates for political explanations and reviews research framing poverty as a political problem and a matter of distribution. Political explanations focus on poverty, not the poor. Political explanations emphasize the essential role of social policy generosity, political choices to penalize risks, power resources, and institutions. Because political explanations provide a better explanation of the causes of the systemically high U.S. poverty, political explanations are far more likely to lead to effective policies.

To conclude, this review is far from the first to criticize the individualistic problem of persons. Historians and critics of American poverty research have long challenged its individualism and argued for structural and political perspectives (*1*–*6*, *15*, *93*, *96*). Readers may recognize that the themes in this review reflect a long-standing critical undercurrent in and the contested nature of perennial poverty debates (*76*). What this review documents, however, is the emergence and ascendance in empirical evidence of systemically high U.S. poverty and its underlying political causes. Political explanations have greater potential and should be prioritized because they have proven more effective at explaining the systemically high U.S. poverty. In turn, this review has aimed to chart a long-term, albeit long overdue and still emerging, justified paradigmatic shift in the social science of poverty. Hence, this review aims to crystalize an ascendant theme to focus on political explanations and poverty, not the poor.
